# Detection of Soluble Antigen and DNA of *Trypanosoma cruzi* in Urine Is Independent of Renal Injury in the Guinea Pig Model

**DOI:** 10.1371/journal.pone.0058480

**Published:** 2013-03-08

**Authors:** Yagahira E. Castro-Sesquen, Robert H. Gilman, Verónica Yauri, Jaime Cok, Noelia Angulo, Hermes Escalante, Caryn Bern

**Affiliations:** 1 Laboratorio de Investigación en Enfermedades Infecciosas, Universidad Peruana Cayetano Heredia, Lima, Peru; 2 Department of International Health, Bloomberg School of Hygiene and Public Health, Johns Hopkins University, Baltimore, Maryland, United States of America; 3 Facultad de Medicina Veterinaria, Universidad Nacional Mayor de San Marcos, Lima, Peru; 4 Department of Pathology, Hospital Nacional Cayetano Heredia, Lima, Peru; 5 Facultad de Ciencias Biológicas, Universidad Nacional de Trujillo, Trujillo, Peru; 6 Departamento de Investigación y Producción, Centro de Análisis e Investigación Escalabs, Trujillo, Peru; 7 Global Health Sciences, Department of Epidemiology and Biostatistics School of Medicine, University of California San Francisco, San Francisco, California, United States of America; Federal University of São Paulo, Brazil

## Abstract

The diagnosis of Chagas disease in humans is generally limited to the detection of specific antibodies. Detection of *T. cruzi* antigens in urine has been reported previously, but is not used in the diagnosis. In this study, soluble *T. cruzi* antigens and DNA were detected in urine samples and were associated with kidney injury and systemic detection of the parasite. We used 72 guinea pigs infected with *T. cruzi* Y strain and 18 non-infected guinea pigs. Blood, kidney, heart and urine samples were collected during the acute phase and chronic phase. Urine samples were concentrated by ultrafiltration. Antigens were detected by Western Blot using a polyclonal antibody against trypomastigote excretory-secretory antigen (TESA). *T. cruzi* DNA was detected by PCR using primers 121/122 and TcZ1/TcZ2. Levels of *T. cruzi* DNA in blood, heart and kidney were determined by quantitative PCR. *T. cruzi* antigens (75 kDa, 80 kDa, 120 kDa, 150 kDa) were detected in the acute phase (67.5%) and the chronic phase (45%). Parasite DNA in urine was detected only in the acute phase (45%). Kidney injury was characterized by high levels of proteinuria, kidney injury molecule-1 (KIM-1) and urea, and some histopathological changes such as inflammation, necrosis, fibrosis and scarce parasites. The detection of antigens and DNA in urine was associated with the presence of parasite DNA in blood and heart and with high levels of parasite DNA in blood, but not with the presence of parasite in kidney or kidney injury. These results suggest that the detection of *T. cruzi* in urine could be improved to be a valuable method for the diagnosis of Chagas disease, particularly in congenital Chagas disease and in immunocompromised patients.

## Introduction

Chagas disease, a parasitic infection caused by *Trypanosoma cruzi*, is one of the most important public health problems in Central and South America. Although the incidence of infection has decreased in recent years due to improved vector control and blood donor screening, there are 8 to 10 million people still infected and an estimated 14,000 deaths associated with the infection occur each year [1]. Making further progress will necessitate programs to diagnose and enable treatment of those already infected, and to screen infants of infected mothers for congenitally acquired infection.

Current diagnostic options for Chagas disease suffer from critical shortcomings in two areas, tests for response to chronic *T. cruzi* infection and detection of congenital infection early in the first year of life. Anti-*T. cruzi* IgG antibody assays provide the most reliable diagnosis of chronic *T. cruzi* infection, but are poor indicators of cure following antiparasitic treatment, taking years to decades to become negative [2], [3]. Molecular methods are currently under study to demonstrate response to treatment in clinical trials. However, 20 to 80% of individuals with chronic infection have baseline negative results by PCR [4]–[6]. Maximizing PCR sensitivity requires multiple large volume blood specimens and sophisticated laboratory expertise and equipment, and is unlikely to be practical for routine use. IgG serological tests cannot be used to diagnose congenital *T. cruzi* infection until 8–9 months of age, because of transferred maternal antibodies [7]. Molecular methods in neonatal blood are promising for congenital Chagas diagnosis, but as noted, require a fairly advanced laboratory and multiple specimens from infants in their first weeks of life [8]. Assays to demonstrate antigen or DNA fragments in urine are attractive alternatives [9], [10]. These substances should disappear from the urine rapidly with successful treatment. Furthermore, the non-invasive nature of urine collection ensures high acceptability by patients and parents.

Pathogen-derived protein and DNA can be seen in urine sediment when the infectious agent invades the kidney or urinary tract [11], [12], or in the soluble portion when circulating proteins or DNA fragments from live or dying organisms in remote locations are filtered into the urine [13], [14]. In theory, only small fragments of 65 kDa or less should be filtered by intact glomeruli [10], but even in infections with no apparent renal injury, larger proteins have been found [15]. Filtration of molecules depends not only on the molecular weight but also on physicochemical properties; in addition, occult damage to the kidneys or other parts of the urinary system may be present in the absence of obvious signs [9], [10]. Trans-renal DNA represents small soluble fragments of cell-free DNA of 150–200 bp (90–120 kDa) excreted from the bloodstream into the urine by as yet unknown mechanisms which may include renal injury [10].

Soluble *T. cruzi* antigens with molecular weights of 150–160 kDa [15], 100 kDa [16], [17], 90–80 kDa [18], 80 kDa [16], [18]–[21], 70–65 kDa [18], 55–50 kDa [22], 55–45 kDa [18], 55 kDa [21], 50 kDa [17], and 40–35 kDa [18] have been reported in urine from animals and patients with Chagas disease. Although *T. cruzi* antigens in urine were presumed to derive from the systemic circulation [16], [17], amastigote nests have been demonstrated in kidney tissue of humans [23] and animals [24]–[26] and in the bladder of animals [27]. Although apparently rare, renal injury has been reported in human *T. cruzi* infection [28]. Furthermore, the murine model of *T. cruzi* infection is characterized by kidney disease caused by decreased renal blood flow in the acute phase [25] and immune complex glomerulopathy in the chronic phase [26].

We have previously described an experimental *T. cruzi* infection model in guinea pigs that resulted in cardiac pathology similar to that in chronic *T. cruzi* infection in humans [24]. In the same model, parasites were also observed in kidney tissue. This study examines the detection of specific soluble proteins and trans- renal DNA in the guinea pig model, and examines the correlation of DNA and antigenuria with markers of kidney injury.

## Methods

### Ethics Statement

The protocol was approved by the San Marcos University Animal Use and Welfare Committee. All experiments adhered to the Guidelines for Animal Experimentation of the Universidad Nacional Mayor de San Marcos.

### Parasites

Trypomastigotes of *T. cruzi* Y strain were donated by Dr. E. Umezawa, Instituto de Medicina Tropical, Universidade de São Paulo, São Paulo, Brazil. The strain was maintained in LLC-MK_2_ cell culture following published procedures [29].

### Animals and Experimental Infection

We used guinea pig samples from our previous published study [24]. Briefly, we used 90 female Andean guinea pigs weighing 600–700 g (two months old). The animals were sourced from the Pachacamac region of Lima, an area without vector-borne transmission of *T. cruzi*. Prior to parasite inoculation, blood samples were taken from each animal and tested for the presence of anti-*T.cruzi* antibodies and *T.cruzi* DNA, and all were negative for both tests [24]. The animals were fed with special food for guinea pigs (cuyina, Purina), alfalfa and water ad libitum. Seventy-two experimental group (EG) guinea pigs were injected with 10 000 parasites in 100 µl RPMI 1640 medium intradermally in the dorsal lumbar region. Eighteen control group (CG) guinea pigs were injected intradermally with 100 µl RPMI 1640 medium alone.

### Sample Collection and Sacrifice of Animals

Eight EG and two CG animals were sacrificed at each time point: 5, 15, 20, 25, 40, 55, 115, 165 and 365 days post inoculation (dpi). For urine collection animals were anesthetized, external urogenital holes were gently disinfected with 7% hydrogen peroxide, guinea pigs were then placed on a bed of plastic, and urine samples were collected by abdominal pressure or aspirated from the plastic if the animals urinated naturally. Blood samples were collected after cardiac puncture and stored at -20°C until use. After urine and blood collection necropsy procedures were performed. The cardiac tissue and right kidney were removed and fixed in ethanol and 10% formalin in PBS. Urine samples were also collected by puncture of the bladder during necropsy.

### Determination of Infection by *T. cruzi*

Infection with *T. cruzi* was confirmed by microhematocrit technique, PCR from blood or cardiac tissue samples [30], [31] and detection of specific antibodies in serum by TESA-blot [32]. The course of *T. cruzi* infection in these animals was published previously by our group. Briefly, the acute phase (20–56 dpi, characterized by the presence of circulating parasites, high levels of specific IgM and abundant amastigote nests and inflammation in cardiac tissue) is followed by the early chronic phase (115–167 dpi, characterized by negative parasitemia and levels of specific IgM, high levels of specific IgG and mild histopathological changes in cardiac tissue), and finally by the chronic phase (365 dpi, characterized by negative parasitemia and low or negative levels of specific IgM, high levels of specific IgG and fibrosis and mild to moderate inflammation in cardiac tissue) [24].

### Treatment of Urine Samples

Approximately 3–10 ml of urine was collected from each animal. Urine samples were immediately centrifuged at 800 g for 20 min, and then the supernatant was stored at −20°C until use. Before concentration, urine samples were again centrifuged and the supernatant was concentrated at 80x by ultrafiltration using Minicon CS15 (Millipore, USA), cut off 15 kDa. The concentrated samples were stored at −20°C.

### TESA Antigen

Trypomastigote excretory-secretory antigen of *T. cruzi* (TESA) was obtained as described previously [29]. It has been reported that serum from patients with Chagas disease recognize two band patterns using the TESA antigen from *T. cruzi* Y strain. The band patterns are: a.) Six bands in a ladder at 130–160 kDa designated as Shed Acute Phase Antigen (SAPA) bands and, b.) A broad antigen band at 150–160 kDa. The 150–160 kDa band is a diagnostic indicator of chronic *T. cruzi* infection [32].

### Production of Antibodies Anti-TESA Antigen

The 150–160 kDa band from the TESA antigen was visualized by Western blot and cut from the nitrocellulose membrane. Two rabbits were immunized intradermally with the 150–160 kDa band (protein concentration 1 mg/ml) emulsified with Freund’s adjuvant (Sigma). This polyclonal antibody recognized a 150–160 kDa band as well as bands between 65 to 85 kDa in total preparations of the TESA antigen. The bands with lower molecular weight could be antigens which share epitopes with the 150–160 kDa band, or degradation products of the 150–160 kDa band. The rabbit immune serum was absorbed with 1/10 normal guinea pig serum in 5% nonfat milk in PBS during 2 hours before use in antigen detection procedures.

### Urine Antigen Detection by Western Blot

Antigens in urine samples were detected as previously described [15]. Briefly, concentrated urine samples were treated with 10% SDS, 5% dithiotreitol, 10% glycerol and 0.01% bromophenol blue and heated to 95°C for 5 min. The antigens were separated on polyacrylamide gels at 10% and, then were transferred to nitrocellulose membranes (BioRad Laboratories). The membranes were incubated with 5% nonfat milk in PBS 0.3% Tween 20 for 1 hour and then with 1/50 absorbed rabbit polyclonal antibody anti-TESA. The antigens were incubated with 1/2 000 peroxidase conjugated goat anti-rabbit IgG (KPL laboratories) for 90 minutes. The antigen-antibody complexes were detected after incubation with 0.5 ug/ml 3,5- diaminobenzidine (Sigma, USA) and 0.03% H_2_O_2_. The molecular weight was determined using Broad Range Standards (BioRad Laboratories). After serial dilutions of TESA antigen in concentrated urine samples, we determined a sensitivity of 0.01 µg of TESA antigen/ml of urine.

### DNA Extraction

DNA was purified by proteinase K digestion (Invitrogen, Carlsbad, CA) and phenol-chloroform extracted as previously described [24], from 500 µl of clot or 25 mg of tissue stored in ethanol. DNA extraction from urine samples was developed according to published protocols with some modifications [24]. Briefly, 25 µl of concentrated urine samples were incubated with 10 mmol/L Tris HCl, pH 7.6, 10 mmol/L NaCl, and mixed gently for 5 minutes. SDS and Proteinase K (Invitrogen, Carlsbad, CA) were added to reach concentrations of 0.25% and 0.50 mg/mL, respectively, and the specimens were incubated for 1 hour at 56°C. DNA was extracted following a standard phenol-chloroform extraction protocol and ethanol precipitation. DNA was suspended in 100 µL Tris HCl, 10 mmol/L, and EDTA 1 mmol/L for clot and tissue samples or 10 µl of the same buffer for urine samples. The quantification of DNA was determined by spectrophotometry using a Nanodrop 2000 instrument (Thermo Scientific, Delaware, USA) and only samples with a ratio of 260 nm/280 nm of ∼1.8 were used for PCR analysis.

### Polymerase Chain Reaction

A PCR targeting the kinetoplast or nuclear DNA of *T. cruzi* was performed as previously described using primers 121/122 (kinetoplast DNA, 330 bp) [30], [31] and TcZ1/TcZ2 (nuclear DNA, 188 bp) [33]. As internal control primers, we used a primer set specific for guinea pig genome Short Interspersed Elements (SINEs) DNA, of which there are an estimated 200–3000 copies in the guinea pig genome [34].

### Real Time PCR

Quantitative real-time PCR in blood and tissue samples was performed using published methods [35] with some modifications [8]. The primer set Cruzi 1 (5–ASTCGGCTGATCGTTTTCGA–3) and Cruzi 2 (5–AATTCCTCCAAGCAGCGGATA–3) were used to amplify a 166–base pair DNA fragment. The probe Cruzi 3 (5–CACACACTGGACACCAA–3) was labeled with 5 FAM (6-carboxyfluorescein) and 3 MGB (minor groove binder). The threshold cycle was determined by the respective standard curve for the specimen batch and was always between 37 and 38 cycles. A specimen was inoculated with 1×10^6^ *T. cruzi* Y strain trypomastigotes, extracted, and diluted successively to determine the minimum quantity detectable; the limit was found to be 1 parasite/ml.

### Urea and Creatinine Levels in Serum

Levels of urea and creatinine in serum were determined by kinetic UV methods (Wiener Lab Group, Argentina). The limits of detection for urea and creatinine were 0.056 mg/dl and 0.01 mg/dl, respectively. Urea and creatinine levels were determined in serum specimens from all non-infected guinea pigs. The normal range was defined as the mean of these values +2 standard deviations.

### Proteins and Kidney Injury Molecule-1 Levels in Urine

Levels of proteins in urine samples were determined by Bradford technique (BioRad Laboratories, USA). The levels of KIM-1 in urine samples were determined by the ELISA test Guinea Kidney Injury Molecule 1 ELISA Kit (MyBiosource, USA), the sensitivity of this assay was 1.0 pg/ml. KIM-1 levels were determined in serum specimens from all non-infected guinea pigs. The normal range was defined as the mean of these values +2 standard deviations.

### Histopathological Analysis

Kidney samples fixed in 10% formalin-PBS were processed and embedded in paraffin. Four 3 µm sections were prepared for each animal: two were stained with hematoxylin-eosin stain and two with Masson’s Trichromic stain. All sections were of approximately equal in size (1 cm). Two entire sections stained by H&E were analysed to determine parasite presence in tissue, and the observation of one or more amastigote nests was considered to be positive. Other pathologic changes such as inflammation, vasculitis, necrosis and fibrosis were observed.

### Statistical Methods

The association between the presence of *T. cruzi* in urine (proteins and trans-renal DNA) with the presence of *T. cruzi* in blood, heart and kidney, and renal injury was determined using logistic regression in STATA10, p values less than 0.05 were considered significant.

## Results

The kinetic profile of *T. cruzi* infection in the guinea pig model was previously defined based on parasitemia, antibody response and histopathological changes, and divided into the following clinical phases: prepatent period (5 dpi), acute phase (15–55 dpi), early chronic phase (115–165 dpi) and late chronic phase (365 dpi) [24].

### Antigen Detection in Urine Samples

**Figure 1:**
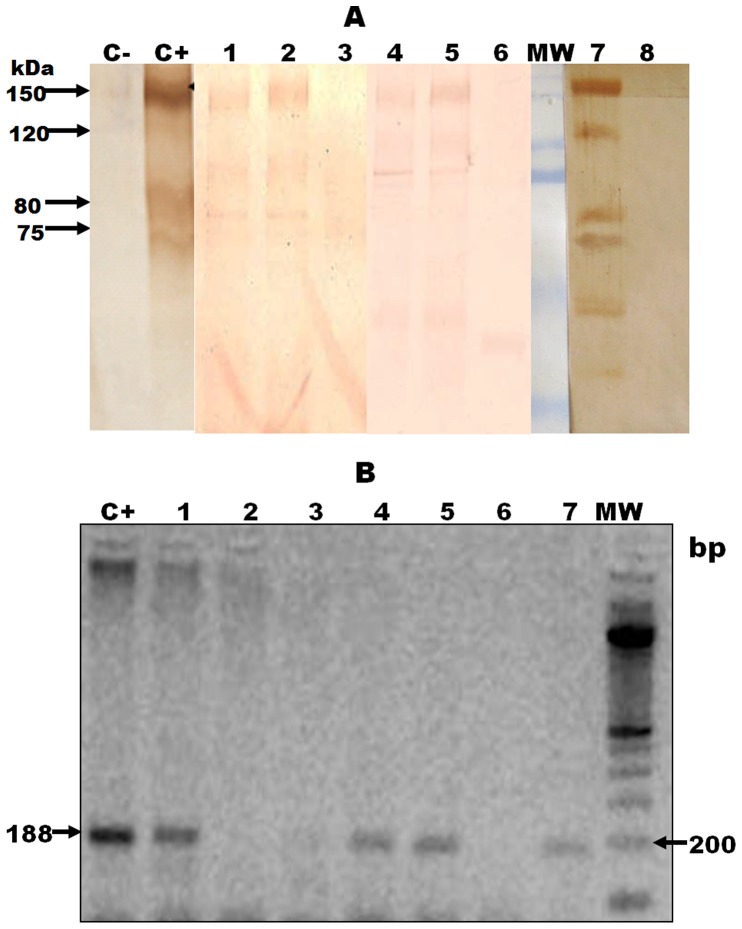
Detection of antigens and DNA of *T. cruzi* in urine of guinea pigs experimentally infected. 1.A. Antigenic bands in urine samples of guinea pigs infected with *T. cruzi*. Bands were detected by Western Blot using a polyclonal antibody against excretory-secretory trypomastigote *T. cruzi* antigen (TESA). C-: Negative control (RPMI 1640 medium). C+: Positive control (TESA antigen). MW: molecular weight marker. Urine samples of infected guinea pigs: Lane 1) 165 dpi, lane 2) 25 dpi, lane 4) 115 dpi, and lane 5 and 7) 55 dpi. Urine samples of non- infected guinea pigs: Lanes 3, 6 and 8. Bands under 70 kDa were considered unspecific because 25% of the non-infected guinea pigs had a reaction to these low bands. 1. B. Detection of trans-renal DNA in urine samples of guinea pig infected with *T. cruzi*. Bands were detected by PCR using primers TcZ1/TcZ2. C+: Positive control (DNA of *T. cruzi* from medium culture). MW: molecular weight marker. Urine samples of infected guinea pigs: Lane 1) 25 dpi, lane 3) 55 dpi, lane 4) 40 dpi, lane 5) 55 dpi, and lane 7) 25 dpi. Urine samples of non- infected guinea pigs: Lanes 2 and 6.

Antigenuria was detected from 20 dpi to 365 dpi. Four bands of 75 kDa, 80 kDa, 120 kDa and 150 kDa were detected in the urine of infected animals (Figure 1); urine samples of control animals were negative throughout the experiment. The 75 kDa and 80 kDa bands were detected from 20 to 365 dpi, whereas the 120 kDa and 150 kDa bands were detected from 25 dpi until 365 dpi. The percentage of animals with antigenuria was higher during the acute (67.5%, 27/40) than chronic phase (45%, 9/20) (p = 0.046) (Table 1).

**Table 1:** Kinetics of antigen detection in urine samples of guinea pig infected with *Trypanosoma cruzi*.

Bands		75 kDa		80 kDa		120 kDa		150 kDa	
Days pi	N	n	%	n	%	n	%	n	%
15	8	0	0.0	0	0.0	0	0.0	0	0.0
20	8	7	75.0	7	100.0	0	0.0	0	0.0
25	8	8	100.0	8	100.0	3	37.5	3	37.5
40	8	6	100.0	6	100.0	2	25.0	2	25.0
55	8	6	75.0	6	75.0	3	37.5	3	37.5
**TOTAL**	**40**	**27**	**67.5**	**27**	**67.5**	**8**	**20.0**	**8**	**20.0**
115	8	2	25.0	2	25.0	4	50.0	4	50.0
165	8	2	25.0	2	25.0	4	50.0	4	50.0
365	4	1	33.3	1	33.3	1	33.3	1	33.3
**TOTAL**	**20**	**5**	**25.0**	**5**	**25.0**	**9**	**45.0**	**9**	**45.0**

Bands were detected by Western Blot using a polyclonal antibody against excretory-secretory trypomastigote *T. cruzi* antigen.

N: Number of animals per group.

n: Number of animals with antigenuria test positive.

%: Percentage of positive animals.

The antigenuria test was considered positive when any of the bands of 75, 80, 120 or 150 kDa were detected.

### DNA Detection in Urine Samples

The mean concentration of cell-free DNA was 47 ng/µl (range 13.4–55.1 ng/µl). In urine from acutely infected animals, both nuclear (188 bp; 18/40 (45%) and kinetoplast (330 bp; 8/40, (20%)) DNA were detected. Urine samples from non-infected guinea pigs and chronic infected animals were negative (Figure 1).

### Detection of *T. cruzi* Antigens and DNA in Urine Samples and Systemic Detection of the Parasite

**Figure 2:**
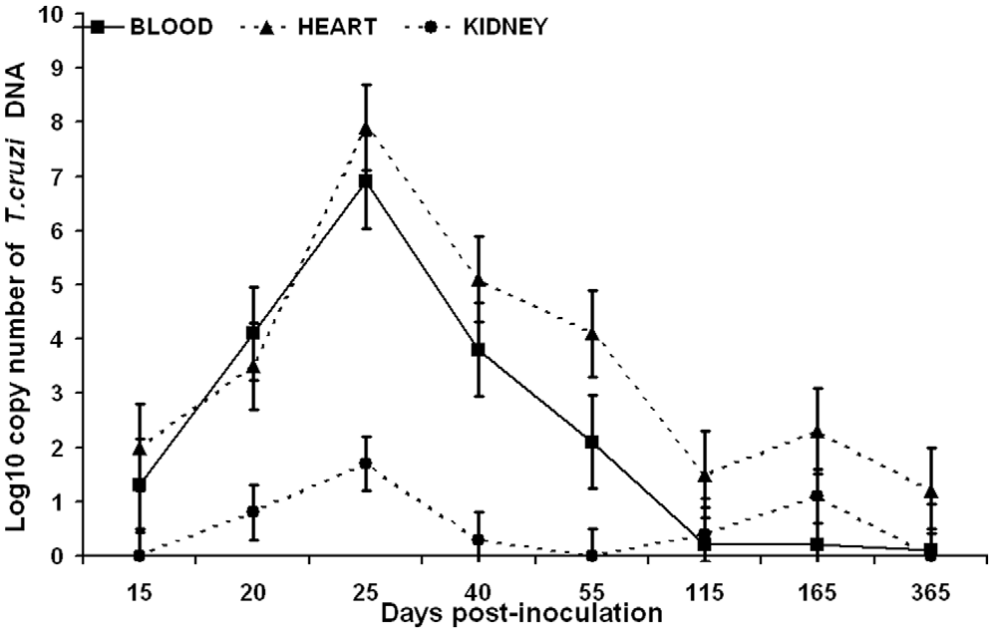
Levels of *T. cruzi* DNA in blood, heart and kidney during the course of infection. Levels of copy number of *T. cruzi* DNA were detected by quantitative PCR. Values represent means and bars represent the standard deviation. Levels of DNA were calculated per ml and mg in blood and tissue, respectively. Eight animals were evaluated in each time point.

Parasite DNA was detected in 100% of blood and cardiac tissue samples during the acute phase with high levels of parasitemia as indicated by high copy numbers of *T. cruzi* DNA; but percentages of detection decreased in the chronic phase (41.6%, 10/24 in blood and 75%, 18/24 in cardiac tissue) (Table 2) (Figure 2). Assays to detect nuclear and kinetoplast DNA were equally sensitive in blood and cardiac samples. RT-PCR showed high levels of parasite DNA during the acute phase in blood and cardiac tissue, with a peak at 25 dpi. These levels decreased after 40 dpi and remained low through the chronic phase (Figure 2). All animals with antigen and/or DNA in their urine also had *T. cruzi* DNA detected in blood and cardiac tissue (Table 2). Detection of antigen and DNA in urine was statistically correlated with high levels of copy number of *T. cruzi* DNA in blood (p = 0.03). No statistical correlation was found between antigen detection and levels of *T. cruzi* DNA in heart and kidney.

**Table 2:** *T. cruzi* antigen and DNA in urine and relationship with systemic detection of the parasite.

			PCR (Blood)^a^		PCR (Cardiac)^b^	
			Positive	Negative	Positive	Negative
Antigenuria^c^	Acutephase^e^	Positive	27	0	27	0
		Negative	13	0	13	0
		Total	40	0	40	0
	Chronicphase^f^	Positive	9	0	9	0
		Negative	1	10	9	2
		Total	10	10	18	2
Trans-renalDNA^d^	Acutephase^e^	Positive	18	0	18	0
		Negative	22	0	22	0
		Total	40	0	40	0
	Chronicphase^f^	Positive	0	0	0	0
		Negative	10	10	18	2
		Total	10	10	18	2

a PCR from blood samples are the results obtained using primers TcZ1/TcZ2.

b PCR from cardiac tissue are the results obtained using primers TcZ1/TcZ2.

c A sample was considered to be antigenuria positive when we detected any of the bands of 75 kDa, 80 kDa, 120 kDa and 150 kDa.

d Trans-renal DNA are the results obtained using the primers TcZ1/TcZ2.

e Number of animals in acute phase = 40.

f Number of animals in chronic phase = 20.

### Kidney Injury and *T. cruzi* Infection

**Figure 3:**
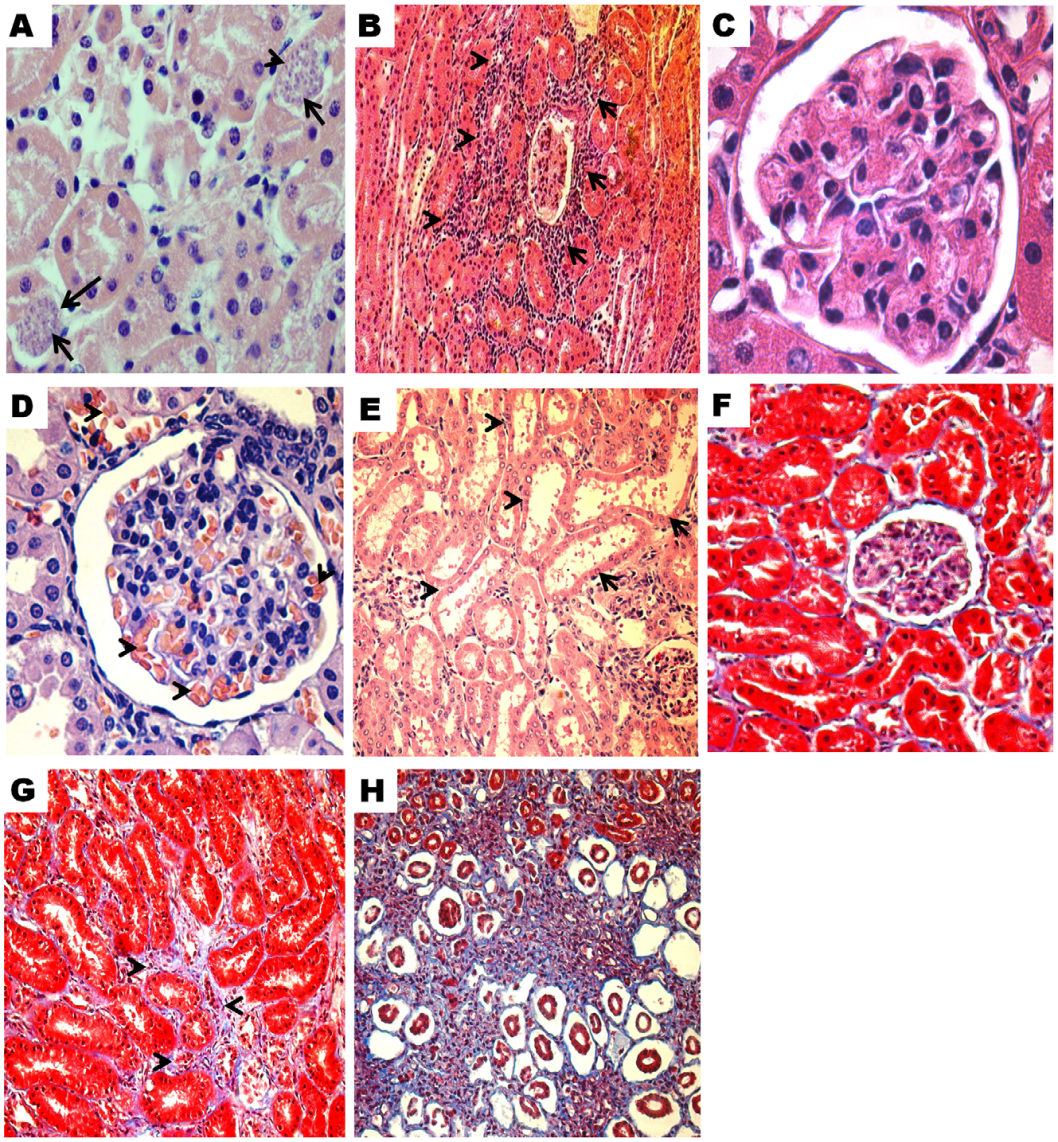
Histopathological changes in kidney tissue of guinea pigs infected with *T. cruzi*. Hematoxylin-eosin stain: A). Amastigote nests (arrows) and tubular necrosis, 25 dpi (500x). B). Focal and mild periglomerular and interstitial inflammation (arrows), 25 dpi (100x). C). Glomerulus of non-infected guinea pig. Note the number of nucleus (1 to 3) in the mesangium (1000x). D). Mesangial hypercellularity and congestion (arrows), 365 dpi (400x). E). Dilatation of proximal tubules (arrows) and periglomerular inflammation, 40 dpi, (200x). **Masson’s Trichromic stain:** F). Kidney tissue of non-infected guinea pig (400x). G). Mild increase in interstitial collagen, 365 dpi (400x). H). Moderate increase in interstitial collagen and tubular atrophy, 25 dpi (200x).

**Figure 4:**
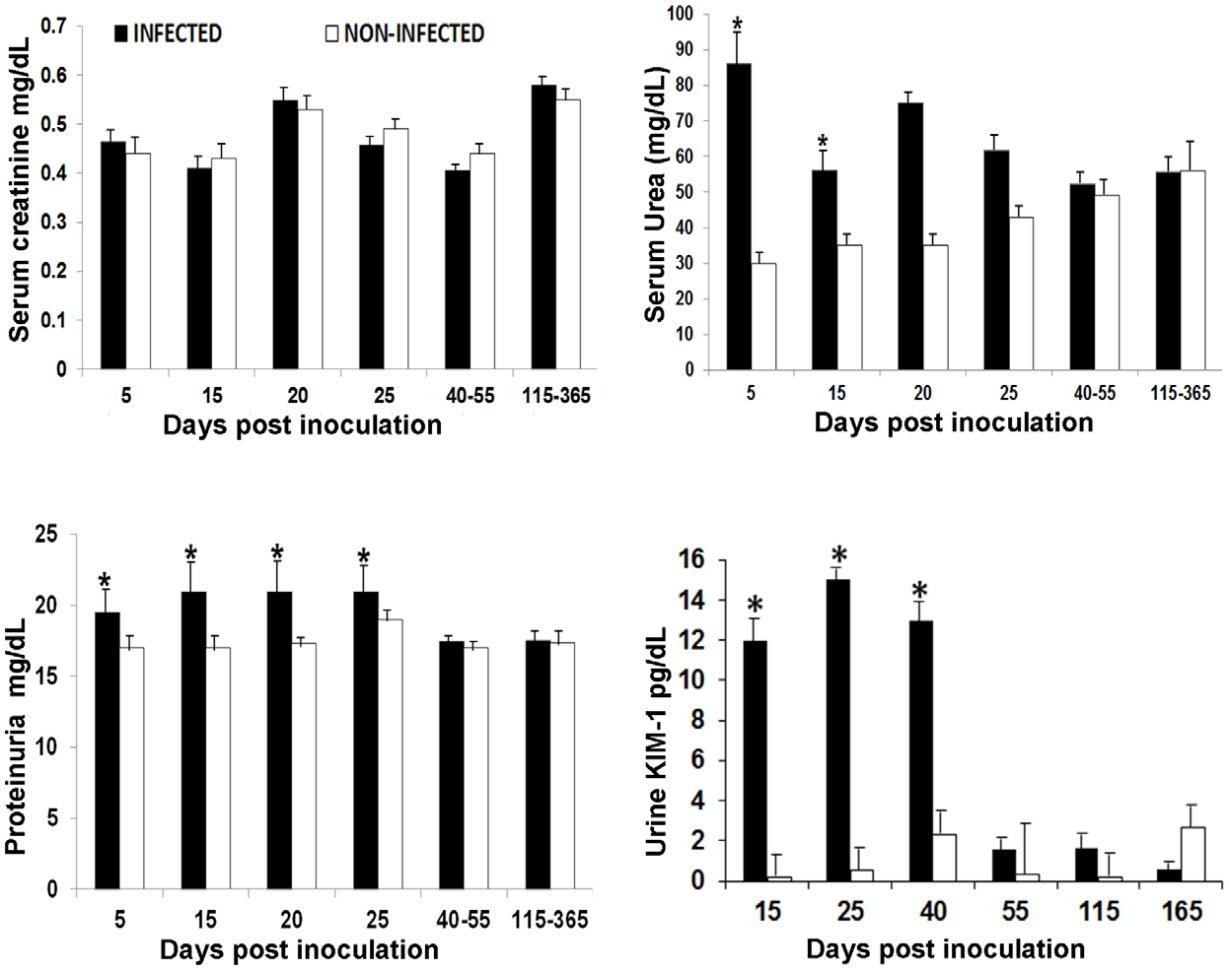
Levels of serum creatinine, serum urea, urine protein and urine KIM-1 in guinea pigs. Bars represent mean values per group; lines on the bars represent the standard deviation * Statistically significant (p<0.05). Number of animals in infected group: 5 days = 8, 15 days = 8, 20 days = 8, 25 days = 8, 40–55 days = 16, 115–365 days = 19. Number of animals in non-infected group: 5 days = 2, 15 days = 2, 20 days = 2, 25 days = 2, 40–55 days = 4, 115–365 days = 6.

Histopathological analysis showed amastigote nests in the proximal and distal tubules in 23.43% (15/64) of infected animals, but the quantity was rare or scarce (1–2 amastigotes nests) (Figure 3a). Kinetoplast or nuclear parasite DNA was detected in kidney tissue of infected animals from 20 dpi until 365 dpi. The percentage of animals with parasite DNA in kidney was slightly higher in the chronic than the acute phase (41.7% (10/24) vs 35% (14/40); p = 0.29) (Table 3). No relationship was found between antigenuria or parasite DNA in urine and parasite DNA detected in the kidney (p>0.05) (Table 4). Histopathological changes in the kidney tissue from infected animals included interstitial inflammation in the tubules and glomerulus (35.0% in acute, 33.3% in chronic phase), congestion (35.0%, 33.3%), vasculitis (22.5%, 16.7%), necrosis in the tubules (35.0%, 20.8%), mesangial hypercellularity (35.0%, 41.7%) and mild fibrosis (22.5%, 20.8%) (Table 3) (Figure 3). Levels of serum urea (32.5%, 13/40), urine proteins (37.5%, 15/40) and KIM-1 (47.5%, 19/40) above the normal range were significantly more common during acute phase infection compared to controls (p<0.05). No elevations in serum creatinine levels were observed in any phase of the infection (Figure 4). There was not significant association between *T. cruzi* antigenuria or DNA in the urine and renal histopathology or biochemical abnormality (Table 4).

**Table 3:** Histopathological and biochemical changes during kidney injury in *C. porcellus* infected with *T. cruzi.*

	ACUTE PHASE (15–55 dpi, n = 40)	CHRONIC PHASE (115–365 dpi, n = 24)
Parasite in Kidney^a^	35.0% (14)	41.7% (10)
Inflammation	35.0% (14)	33.3% (8)
Congestion	35.0% (14)	33.3% (8)
Vasculitis	22.5% (9)	16.7% (4)
Mesangial hypercellularity	35.0% (14)	41.7% (10)
Fibrosis	22.5% (9)	20.8% (5)
Necrosis	35.0% (14)	20.8% (5)
Biochemical alterations^b^	47.5% (19)	12.5% (3)

a Parasites in kidney were detected by H&E and PCR. In order to be considered positive one or more parasites needed to be observed in two entire tissue sections.

b Corresponds to the increase of level of two or more biochemical parameters: serum urea, urine proteins and urine KIM-1.

n = Number of animals analyzed.

**Table 4:** Detection of *T. cruzi* antigen and DNA in urine samples and kidney injury during infection.

		POSITIVES FOR ANTIGENURIA AND:				
	Antigenuria^a^	Parasite in kidney^b^	Histopathological Changes^c^	BiochemicalAlterations^d^		Histopathological and Biochemical changes
Acute Phase^e^	27	9 (p = .065)	12 (p = .302)	9 (p = .098)		9 (p = .095)
Chronic Phase^f^	9	2 (p = .375)	2 (p = .107)	1 (p = .100)		1 (p = .120)
		**POSITIVES FOR TRANS-RENAL DNA AND:**				
	**Trans-renal DNA** g	**Parasite in kidney** b	**Histopathological Changes** c	**Biochemical Alterations** d	**Histopathological and Biochemical changes**	
Acute Phase^e^	18	7(p = .641)	7 (p = .135)	5 (p = .207)	5 (p = .207)	

a Corresponds to the detection of bands of 75, 80, 120 and/or 150 kDa.

b Parasites in kidney were detected by H&E stain or PCR. In order to be considered positive one or more parasites had to be observed in two entire tissue sections.

c Observation of three or more of the following histopathological changes: inflammation, necrosis, fibrosis, vasculitis, congestion, fibrosis and mesangial hypercellularity.

d Increase in level of two or more biochemical parameters: serum urea, urine proteins and urine KIM-1.

e Acute phase: n = 40 samples analyzed.

f Chronic phase: n = 19 samples analyzed.

g Results obtained using primers TcZ1/TcZ2.

## Discussion

Our most significant finding was that a large proportion of animals with acute infection and a smaller proportion in the chronic phase excreted detectable antigen in the urine, while trans-renal DNA was seen in the acute phase only. Although we found histopathological and biochemical evidence of renal damage, the changes were generally mild, consisting of glomerulitis, and focal tubular inflammation, and no association was seen between kidney damage and urinary antigen or DNA. These findings raise the possibility that if the sensitivity of antigen and/or DNA detection in urine improves, this approach could be adapted to develop new diagnostic tests for congenital *T. cruzi* infection and to monitor response to anti-trypanosomal treatment.

Our observations are consistent with previous publications demonstrating *T. cruzi* antigens in urine from animals and patients [15]–[18], [21]. Urinary *T. cruzi* antigens have been shown to correspond to glycoproteins [16], proteins belonging to the transferrin family [20], parasite tubulin [22] or cruzipain C-terminal extension [36]. However, these observations have never been widely exploited for the diagnosis of Chagas disease. We detected four antigens of 75 kDa, 80 kDa, 120 kDa and 150 kDa in urine of infected animals.

The sensitivity of antigen detection in our infected guinea pigs (67.5% in acute phase and 47.4% in the chronic phase) is lower than reported previously; other studies have shown sensitivity of 100% in acute [18], [21] and 32.6%–93% in chronic patients [15]–[18]. This higher sensitivity may be related to the higher sample volume that can be obtained from human patients (50 ml to 300 ml), compared to the guinea pig (3–10 ml). Another explanation for the lower sensitivity could be the antibody used for antigen detection [15]–[22]. Furthermore, the presence of proteases, temperature, and pH that affect the stability of proteins in urine [37], [38] could also decrease the sensitivity of antigen detection, especially in those samples with low concentrations of antigens.

RT-PCR showed the kinetics of parasite detection in blood and cardiac tissue, with the highest levels during the acute phase and lowest levels during the chronic phase. This is consistent with our previous results using microscopy to detect parasites [24]. One limitation of this study is that we were not able to collect serial urine samples of the same animal to avoid overstressing the guinea pigs. However, the association of antigen detection with high levels of parasite DNA in blood and the lack of association with kidney injury and parasite presence in the kidney strongly suggest that antigens in urine come from systemic circulation.

Parasite DNA was detected in blood from infected animals very early in infection (5 days pi) and in all specimens during the acute phase; however, in the chronic phase, the proportion with positive results fell substantially. This observation is similar to that observed in dogs infected with *T. cruzi* strain Y [39] and to that reported in humans [40], and reflects the steep decline in the level of circulating parasites when the animal enters the chronic phase.

This work constitutes the first report of detection of *T. cruzi* DNA in urine. We were not able to detect *T. cruzi* DNA in urine during the chronic phase, presumably reflecting lower circulating parasite load and the consequent fall in circulating DNA fragments available to cross into the urine. Procedures for maximizing the sensitivity of trans- renal DNA detection are not yet fully established; there is still a need to standardize sampling protocols, treatment, storage, and DNA extraction [10]. Urine samples were concentrated by ultrafiltration, which is a highly recommended methodology for trans-renal DNA concentration and removes salts in urine that can inhibit PCR reactions [10]. The phenol-chloroform protocol for DNA extraction that we used has been shown to remove PCR inhibitors in urine samples [41], and the 260 nm/280 nm ratios confirmed the purity of our samples. However, one limitation of this study is that we did not include DNase inhibitors, high activities of DNase have been demonstrated in urine [42], [43].

Although the origin of trans-renal DNA is still uncertain, *T. cruzi* is known to die by apoptosis leading to internucleosomal DNA fragmentation [44]. Trans-renal DNA is hypothesized to consist of mono- and di-nucleosomes derived from apoptotic cells that cross the renal barrier and appear in urine [45]. The higher sensitivity of nuclear primers over those targeting the kinetoplast is likely due to the size of their respective amplification products (188 vs 330 bp). Cell-free DNA are generally products of 150–200 bp, and lower sensitivity has been reported with larger products [46].

This study shows the evolution of the kidney disease during acute and chronic infection with *T. cruzi* in the guinea pig model. In our study, serum urea, urine proteins and KIM-1 levels increased modestly, in the absence of any change in serum creatinine, reflecting only mild renal damage in the guinea pig model, in contrast to more severe acute renal damage reported in murine models [25], [26]. Creatinine concentrations is reported to increase only when there is a loss of at least 50% of kidney function, demonstrating the utility of KIM-1 in renal injury detection [47]. In our model, kidney injury was not associated with the presence of parasites in the kidney, but was associated with cardiac pathology (p = 0.048), suggesting that cardiac damage, characteristic of infection of guinea pigs with *T. cruzi* [24], affects renal function indirectly, possibly through activation of Renal Angiotensin System (RAS) [25]. The results obtained here show that the guinea pig model can be used for further studies in kidney disease during *T. cruzi* infection.

The lack of association with renal damage indicators suggests that the antigen and DNA found in urine were products released by the parasite into blood or tissues and then excreted in the urine. This phenomenon has been reported in *Mycobacterium tuberculosis* infection in which proteins and DNA are detected in urine of patients with pulmonary disease in the absence of renal involvement, and can be exploited for diagnostic purposes [14], [48]. Nevertheless, origin from other parts of the urinary system cannot be entirely ruled out, since *T. cruzi* can infect the bladder and urinary system [49].

Our results suggest that detection of *T. cruzi* in urine could be developed into a useful tool for the diagnosis of Chagas disease, especially in the acute phase, and if the sensitivity can be improved, in the chronic phase as well. These assays may be especially valuable for the diagnosis of congenital Chagas disease, in immunosuppressed patients and for the evaluation of treatment efficacy. Our findings show that excretion of soluble antigens and DNA in urine results from systemic infection and not directly from kidney involvement. The next steps will be the addition of concentration techniques to improve sensitivity and adaptation of these assays for urinary diagnosis of *T. cruzi* in human specimens, particularly in congenital Chagas disease.
